# 

**Published:** 1899-09-30

**Authors:** 


					.. SPI1AL, jl*4 The Standard of highest purity."? Lancet. Ski-tembeb 30th, 189\
CADBURY'S m U CADBURY'S
COCOA Ik JMC? . COCOA
ABSOLUTELY PURE. *  NO DRUGS OR ALKALIES USED.
No. 679. JVol XXVI. [aRgpreapear3 Saturday, Sept.
30th, 1899. [_'
rSnbsc
ription Terms,J pRICE TWOPENCE.

				

